# Understanding paraxial mesoderm development and sclerotome specification for skeletal repair

**DOI:** 10.1038/s12276-020-0482-1

**Published:** 2020-08-13

**Authors:** Shoichiro Tani, Ung-il Chung, Shinsuke Ohba, Hironori Hojo

**Affiliations:** 1 Sensory & Motor System Medicine, Graduate School of Medicine, The University of Tokyo, Tokyo, 113-8655 Japan; 2 Center for Disease Biology and Integrative Medicine, Graduate School of Medicine, The University of Tokyo, Tokyo, 113-8655 Japan; 3 Department of Bioengineering, Graduate School of Engineering, The University of Tokyo, Tokyo, 113-8656 Japan; 4 Department of Cell Biology, Institute of Biomedical Sciences, Nagasaki University, Nagasaki, 852-8588 Japan

**Keywords:** Pluripotent stem cells, Bone development

## Abstract

Pluripotent stem cells (PSCs) are attractive regenerative therapy tools for skeletal tissues. However, a deep understanding of skeletal development is required in order to model this development with PSCs, and for the application of PSCs in clinical settings. Skeletal tissues originate from three types of cell populations: the paraxial mesoderm, lateral plate mesoderm, and neural crest. The paraxial mesoderm gives rise to the sclerotome mainly through somitogenesis. In this process, key developmental processes, including initiation of the segmentation clock, formation of the determination front, and the mesenchymal–epithelial transition, are sequentially coordinated. The sclerotome further forms vertebral columns and contributes to various other tissues, such as tendons, vessels (including the dorsal aorta), and even meninges. To understand the molecular mechanisms underlying these developmental processes, extensive studies have been conducted. These studies have demonstrated that a gradient of activities involving multiple signaling pathways specify the embryonic axis and induce cell-type-specific master transcription factors in a spatiotemporal manner. Moreover, applying the knowledge of mesoderm development, researchers have attempted to recapitulate the in vivo development processes in in vitro settings, using mouse and human PSCs. In this review, we summarize the state-of-the-art understanding of mesoderm development and in vitro modeling of mesoderm development using PSCs. We also discuss future perspectives on the use of PSCs to generate skeletal tissues for basic research and clinical applications.

## Regenerative medicine: the challenge of building skeletal tissue

A deeper understanding of skeletal tissue development and improvements in tissue engineering will help pluripotent stem cell (PSC) therapies to reach their full potential for skeletal repair. The paraxial mesoderm, an embryonic germ layer, is crucial to the formation of healthy axial skeleton. Shoichiro Tani at the University of Tokyo, Japan, and co-workers reviewed current understanding of paraxial mesoderm development and studies involving in vitro PSC skeletal modeling. The formation of the paraxial mesoderm and associated connective tissues comprises multiple stages, and studies in vertebrate embryos have uncovered critical signaling pathways and cellular components important to PSC modeling. Although many individual cellular components can now be modeled, it remains challenging to recreate three-dimensional skeletal tissues. Such an achievement would facilitate a functioning model of bone metabolism, the next step in achieving skeletal regeneration.

## Introduction

Although the continued elongation of life expectancy is generally a positive development for mankind, it also has certain unintended consequences, including the numerous social and medical problems associated with an aging society. Prominent among these problems are skeletal disorders, such as sarcopenia, osteoporosis, fragile bone fractures, and osteoarthritis. Bone formation and resorption, i.e., bone remodeling, dynamically occur in the whole body throughout the whole life span^1^. Although bone has the potential to repair itself, this ability is limited. Thus, trauma and skeletal disorders in elderly individuals cause critical unrepaired bone defects, resulting in a compromised quality of life^2^.

Because there are remaining unmet needs that cannot be solved by the conventional treatments of skeletal disorders, regenerative medicine has been attracting attention for the past several decades^3^. The emerging technology of induced pluripotent stem cells (PSCs) has expanded our strategies in both basic research and clinical applications, enabling the modeling of human development and genetic diseases, the establishment of drug screening systems, and clinical trials for the transplantation of differentiated cells into patients^4,5^. In this review, we focus on the paraxial mesoderm, which is one of the main origins of skeletal tissues. We summarize the latest understanding of mesoderm development, which is mainly based on studies in mammals and birds. We also introduce recent studies concerned with the in vitro modeling of mesoderm development using PSCs, and discuss future perspectives on the use of PSCs for basic research and clinical applications.

## Overview of bone development

Bones originate from three types of cells: the paraxial mesoderm, lateral plate mesoderm, and neural crest^6^. The paraxial mesoderm gives rise to the axial skeleton. The lateral plate mesoderm gives rise to the appendicular skeleton. The neural crest originates from the ectoderm and gives rise to the craniofacial skeleton^6^.

There are two processes involved in bone formation: intramembranous ossification and endochondral ossification. While endochondral ossification occurs via cartilage formation and is followed by bone formation, intramembranous ossification occurs through direct transition from mesenchymal cells to bone-forming osteoblasts^6^. Although the neural crest forms most of the craniofacial skeleton by intramembranous ossification (with exceptions such as the skull base), most mesoderm-derived bones are formed through the endochondral ossification process^6^. Because of the large contribution of the mesoderm population to skeletal development, we focused on the paraxial mesoderm lineages in this review.

## Development of the mesoderm

### Overview of the mesodermal derivatives (Fig. 1)

The mesoderm initially forms in the primitive streak during gastrulation and later continues developing in the tail bud. First, the chordamesoderm forms a notochord that expands beneath the neural tube, as observed in human embryos^7^. The mesoderm lies along the notochord and divides into the paraxial mesoderm, intermediate mesoderm, and lateral plate mesoderm^8^. Mesodermal subtypes are specified along the mediolateral axis depending on the activities of BMPs^9^. A study with chick embryos demonstrated that Noggin, a BMP inhibitor expressed first in the notochord and then in the somatic mesoderm, creates the BMP gradient that specifies the mesodermal subtypes^10^. Another study with mouse embryos demonstrated that the notochord gives rise to the nucleus pulposus, which later forms vertebral discs^11^.

**Fig. 1:**
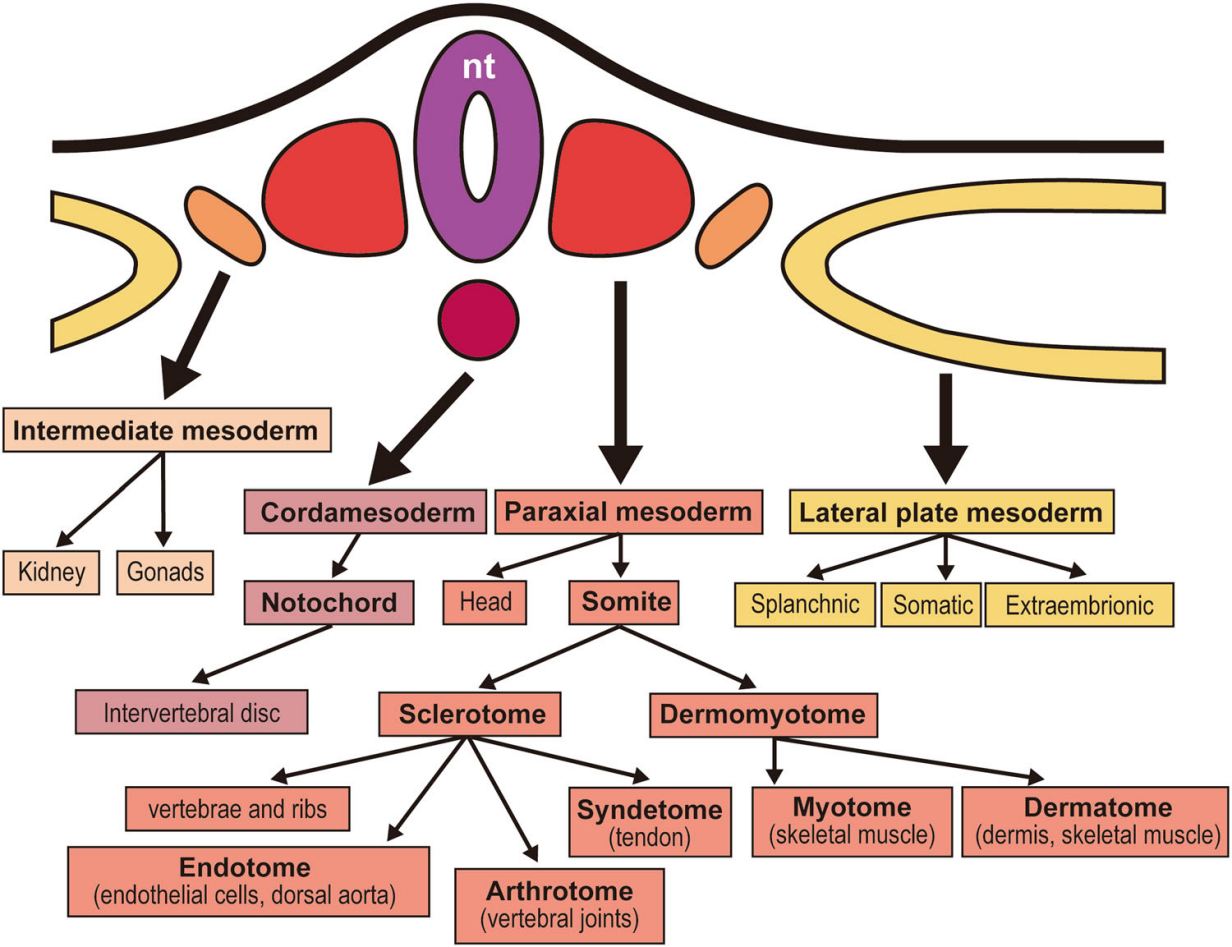
Overview of the mesodermal derivatives. The chordamesoderm and paraxial mesoderm form the axial skeleton, whereas the intermediate mesoderm forms the kidneys and gonads, and the lateral plate mesoderm forms the circulating systems, body wall, and limbs (except for the musculature). nt neural tube. This figure is a modified version of an image from a textbook^107^.

Paraxial mesoderm development is composed of several stages: presomitic mesoderm specification, somitogenesis, and somite specification^12^. Mature somites contain two major populations: the sclerotome and dermomyotome. The sclerotome gives rise to the vertebrae and associated ribs, tendons, and other tissues, such as vascular cells of the dorsal aorta, intervertebral blood vessels, and meninges^12,13^. The dermomyotome produces two components: the myotome and the dermatome. The myotome gives rise to the musculature of the back, rib cage, ventral body wall, and limbs. The dermatome gives rise to the dermis of the back, although the term dermomyotome is sometimes used to describe this region because a recent study showed that this central region of the dermomyotome also gave rise to muscles in chick embryos^14^.

The lateral plate mesoderm forms the splanchnic mesoderm, somatic mesoderm, and extraembryonic membranes, as evidenced by a study of chick embryos^15^. The splanchnic mesoderm gives rise to components of the circulatory system, such as the heart, blood vessels, and blood cells, whereas the somatic mesoderm forms the pelvic skeleton and mesodermal components of the limbs, with the exception of the muscles, which are derived from the dermomyotome^14,16^. The intermediate mesoderm forms the urogenital system, including the kidneys and gonads^8^.

### Specification of the presomitic mesoderm

The early paraxial mesoderm is referred to as the presomitic mesoderm, and consists of bilateral streaks of mesenchymal cells adjacent to the notochord^17^. The presomitic mesoderm is derived from the primitive streak or neuromesodermal progenitors in the tail bud, as shown in studies with mouse and bird embryos^18,19^. In these steps, Noggin produced by the notochord protects the paraxial mesoderm from lateralization by BMPs produced by the intermediate mesoderm and lateral plate mesoderm^9,10^. This gradient is crucial for mesodermal cell fate determination^9,10^. When Noggin-expressing cells were implanted into the presumptive lateral plate region, somitic tissues were formed in the original lateral plate territory of chick embryos^10^. This demonstrates that the paraxial mesoderm and lateral plate mesoderm share common precursors in the primitive streak, and that the cell fate is plastic, depending on the gradients of BMP activity.

Wnt signaling is another crucial pathway in these processes. Wnt3a is widely expressed in the primitive streak and tail bud, as revealed in a study of mice^18^. Loss of function of *Wnt3a* or *Ctnnb1*, which is a gene encoding β-catenin, led to a loss of paraxial mesoderm progenitors and their derivatives, the presomitic mesoderm and somites in the mouse embryos^18^. Key transcriptional regulators in presomitic mesoderm specification, including Brachyury (T), Tbx6, and Mesogenin1 (Msgn1), are known to be downstream factors of Wnt signaling^20^.

T, the T-box transcription factor^21^, is expressed in the primitive streak, the tail bud, the early mesoderm and primitive ectoderm next to the primitive streak, the notochordal plate, and the notochord, as shown by studies of mouse embryos^22^. Classical genetic analyses using spontaneous mutant mice revealed that *T* is essential for mesoderm formation^22,23^. Loss of function of *T* caused a disturbance of primitive streak formation and insufficient mesoderm formation in mouse embryos^23^.

Tbx6, the T-box transcription factor, is expressed initially in the primitive streak, and later in the tail bud and presomitic mesoderm^24^. Studies with mouse embryos demonstrate that *Tbx6* expression in the paraxial mesoderm is restricted to the presomitic mesoderm and is rapidly downregulated as somite forms^24^. Thus, the expression of *Tbx6* overlaps that of *T* in the primitive streak and tail bud, although *T* is expressed at an earlier point in the primitive streak^24^. Loss of function of *Tbx6* in mice resulted in conversion of the presumptive presomitic mesoderm into neural tissues^25^. In *Tbx6*-knockout mice, *Sox2*, a member of the Sry-related high mobility group box containing genes, was ectopically expressed in a presumptive presomitic mesoderm region; *Sox2* was not expressed in that region in wild-type mice^25^. Given that Sox2 is known to be a critical factor for neuroectodermal development, this indicates that Tbx6 promotes presomitic mesoderm specification by repressing *Sox2* expression and neural fates^25^.

Msgn1, the basic helix-loop-helix (bHLH) transcription factor, is expressed in the paraxial mesoderm from gastrulation until somite formation^26^. Overexpression of *Msgn1* in mice expanded *Tbx6*-expressing regions through the trunk, resulting in expansion of the presomitic mesoderm region into the anterior^27^. The loss of function of *Msgn1* reduced *Tbx6* expression in the presomitic mesoderm^27^, and caused a complete failure of somite formation and segmentation of the body trunk and tail in mice^26^. These results indicate that Msgn1 is another determinant of presomitic mesoderm fate specification.

### Specification of neuromesodermal progenitors

In the posterior region of the embryo, the paraxial mesoderm is derived from a cell population called neuromesodermal progenitors, which have the bipotential to differentiate into both mesodermal and ectodermal cell types, as demonstrated in a mouse study^28^. The cell fate is determined by several morphogens expressed along the anterior–posterior axis. Histological studies with mouse embryos demonstrated that Fgf8 and Wnt3a are highly expressed in the vertebrate tail bud, whereas the retinoic acid (RA) gradient is produced from the somite and the neural plate^29^. Fgf8 and Wnt3a were found to upregulate both *Msgn1* and *Tbx6*, resulting in the promotion of presomitic mesoderm specification by suppressing *Sox2* expression and neural cell fate specification in mouse embryos^25,30^.

Loss of function analysis with aldehyde dehydrogenase 1 family member A2 (Raldh2), which functions as a catalyst of RA synthesis, revealed that the Raldh2^−/−^ mouse embryos exhibited increased Fgf8 expression in the anterior part of the embryo^29^. The deficient mice also displayed impaired formation of somites with a decrease in Sox2-positive and Sox1-positive neuroectodermal progeny, and an increase in Tbx6-positive presomitic mesodermal progeny^29^. The phenotype of impaired somite formation in Raldh2^−/−^ mice was rescued by treatment, with an FGF receptor antagonist^29^. These findings indicate that RA directly represses the expression of Fgf8 and Tbx6, resulting in cell fate specification to neural cell types with upregulation of Sox2. They further suggest that the balance of signaling activities between these opposing morphogens is a key determinant of neural and mesodermal cell fates^29,30^.

### Somitogenesis

The somite is derived from the anterior presomitic mesoderm through a series of dynamic morphogenetic events that involve cyclical signaling. The periodicity of somitomere formation is produced by the segmentation clock that operates in the presomitic mesoderm. A study with mouse embryos demonstrated that this segmental prepattern is defined at the “determination front”, which creates future somitic boundaries^31^. This process proceeds according to a “clock and wavefront model”: a clock determines the time, and a wavefront determines the place for the segmentation^32^. Mesenchymal–epithelial transition (MET) is another essential process for somitogenesis, as it is involved in epithelial somite formation^33^. Studies with mouse embryos demonstrate that during these processes, *Msgn1* is downregulated, but several other markers, including *Mesp2*, *Paraxis*, *Pax3*, *Foxc1/2*, and *Meox1/2*, are upregulated^9,34–36^.

#### Segmentation clock

The major signaling pathways in the segmentation clock are the Notch, Wnt/b–catenin, and FGF pathways, which integrate to form a molecular network and generate a traveling wave of gene expression along the embryonic axis. Global gene expression analysis in mice revealed that Notch- and FGF-related cyclic genes oscillated mostly in the opposite phase of the Wnt-cyclic genes, suggesting crosstalk between these signaling pathways^37,38^. In mice, the clock in each region of the presomitic mesoderm is well understood to be a negative feedback mechanism centered on the activities of the transcription factor Hes7^39,40^. *Hes7* is initially activated by FGF signaling, and then it is controlled by Notch activity^40^. Hes7 suppresses its own transcription to generate an oscillating pattern of expression^39^. Notch signaling activates mesodermal posterior 2 (Mesp2), a bHLH transcription factor, which suppresses the Notch pathway via lunatic fringe (*L-fng*)^41^. Thus, Notch activity oscillates in the presomitic mesoderm as a “Notch clock oscillator”^42^. FGF signaling also oscillates via the phosphorylation of extracellular signal-regulated kinase, an FGF signaling downstream molecule, which was also demonstrated in mice^43^.

#### Determination front and segmentation

Mesp2 is a master regulator of the onset of segmentation^35^. *Mesp2* is expressed at the initial stage of segmentation in the presomitic mesoderm^35^, and its expression is restricted to the rostral compartment by the oscillators of the Notch and FGF pathways, as evidenced by a study of mouse embryos^43^. *Mesp2* expression is activated by the Notch pathway in the anterior part of the presumptive somite^44^, whereas it is suppressed by FGF signaling in the posterior part, resulting in the formation of anterior and posterior borders^35,45^. This model is supported by several lines of evidence. First, *Mesp2* expression was strongly suppressed in Notch mutant mouse embryos, such as *Dll1*-null and *RBP-jk*-null embryos^40^. Second, the *Mesp2*-expressing domain was shifted into the posterior presomitic mesoderm in the absence of FGF signaling in mouse embryos^43^.

As described earlier, Mesp2 plays a crucial role in the formation of the border of somite segments and in establishing the rostrocaudal patterning of each somite^35,42^. *Mesp2*-null mouse embryos were shown to have a nonsegmented somite with completely caudalized somite derivatives^35^. Notch activity is required for caudalization of the somite, since its absence in the caudal compartment resulted in a rostralized phenotype in mice^46^. With respect to the mechanism of Mesp2-mediated somite patterning, a study in mouse embryos demonstrated that Mesp2 suppresses Notch activity in the rostral compartment by destabilizing Mastermind-like 1, one of the core components of the nuclear Notch intercellular domain complex^47^. This leads to rostrocaudal formation via differential Notch activity^47^.

A study with mouse embryos demonstrated that Mesp2 activates its target *Ripply2*, which suppresses *Mesp2* expression via the inhibition of *Tbx6* in a negative feedback loop, leading to formation of the next segmental border^48^. Another research in mice has demonstrated that Mesp2 also upregulates *Eph* in the anterior portion of somitomeres, which is followed by the upregulation of *ephrin* in the opposing posterior half of the more anterior somitomere^49^. Then, separation of the somite from the anterior end of the presomitic mesoderm occurs at the border between *ephrin-* and *Eph*-expressing cells^49^. This pattern is repeated sequentially in the process of somitogenesis^42^.

#### Mesenchymal–epithelial transition

MET is necessary to form the epithelial layer of the somite during somitogenesis, since the presomitic mesoderm is composed of only mesenchymal cells. A study with mouse embryos demonstrated that without MET, neither the epithelial somite nor the dermomyotome can properly form; the absence of MET leads to abnormalities of the axial skeleton, such as numerous patterning defects of the musculature in the axial skeleton, limbs, and body wall^33^. During MET in the future somatic boundaries, the outer epithelial layer assumes apical–basal polarity and expresses N-cadherin, β-catenin, and F-actin in apical adherens junctions^50^. This process is intimately regulated in a spatial and temporal manner along the anterior–posterior axis, as evidenced by a study in birds^50^.

Paraxis, a bHLH transcription factor, is expressed in the presomitic mesoderm and somites. Paraxis is indispensable for epithelialization in the developmental process of somite^33^. *Paraxis-*null mice had no epithelial somites, although the somites were segmented into loose mesenchymal units of approximately the correct size and periodicity as somites in the paraxial mesoderm^33^. The mutants also displayed skeletal abnormalities, such as caudal agenesis^33^. These facts suggest that Paraxis is required for the formation of the epithelial somite but not for segmentation of the paraxial mesoderm.

Somitic MET in the mouse and chick paraxial mesoderm is dependent on Wnt signaling from the overlying surface ectoderm^51,52^. Although segmentation of the paraxial mesoderm was maintained even with the removal of the surface ectoderm, somitic MET did not occur in mice^52^. Loss of Wnt signaling caused loss of Paraxis expression and somitic MET in mice^52^. In addition, Wnt6 expression in the surface ectoderm induced somitic MET, and ectopic Wnt6 expression substituted for a lack of surface ectoderm and β-catenin dependent processes in chick embryos^53^. Moreover, forced expression of paraxis rescued somite epithelialization in the absence of Wnt signaling in chick embryos^54^. On the other hand, *paraxis*^−/−^ mouse embryos showed decreased expression of downstream genes in the Wnt and Notch signaling pathways, as well as decreased expression of Meox1/2 and Pax1, which are required for proper somite formation and specification, respectively^55^. These facts suggest that paraxis participates in Wnt signaling-mediated epithelialization in PSM^55^.

A previous report demonstrated that Meox1 and Meox2 are coexpressed in the epithelial somites, sclerotome, and limb buds, whereas the dermomyotome only expresses Meox1^56^. *Meox1*-null mutant mice had defects in axial skeletal development but not muscle development^57^, whereas *Meox2*-null mutant mice displayed a lack of limb muscles, as well as a generally reduced muscle mass, but no abnormality in the axial skeleton^56^. These results suggest that Meox1 substitutes for Meox2 in the sclerotome, but not the myotome and that Meox2 compensates for the lack of Meox1 in the myotome, but not the sclerotome.

Foxc1 and Foxc2, members of the winged helix transcription factors, are expressed in many tissues forming the somites, head mesoderm, and endothelial and mesenchymal cells of the developing heart and blood vessels, as shown in mouse embryos^36^. Mouse embryos lacking both *Foxc1* and *Foxc2* had no epithelial somite or morphological segmentation of the paraxial mesoderm^36^. *Paraxis* was undetectable in the presomitic mesoderm and somite region in the mutant, suggesting that Foxc1 and Foxc2 are upstream of paraxis during the somite formation processes^36^. Another report showed that the paraxial mesoderm in Foxc1/2 mutant mice was respecified into the intermediate mesoderm, which expresses Pax2; Pax2 is a major transcription factor in the intermediate mesoderm^58^. However, no significant change was detected in the expression of either Bmp4 or Noggin, which can regulate mesodermal fates^58^. In addition, misexpression of Foxc1 or Foxc2 in the presumptive intermediate mesoderm of mouse embryos resulted in conversion of the cell fates from the intermediate to paraxial mesoderm and somite, but not to the lateral plate mesoderm^58^. These results suggest plasticity of the cell fates between the intermediate mesoderm and the paraxial mesoderm, with Foxc1 and Foxc2 contributing to somite segmentation in the paraxial mesoderm. Taken together, these results show that Foxc1 and Foxc2 are essential for paraxial mesoderm differentiation and fate determination.

### Specification of the somite

The sclerotome is derived from a ventromedial part of the somite and is formed by epithelial–mesenchymal transition, whereas the dermomyotome is derived from the epithelial dorsolateral part of the somite^59^. The sclerotome is a mesenchymal tissue in which key regulators, including Pax1, Pax9, Nkx3.2 (Bapx1), and Sox9, are specifically expressed^60^. On the other hand, Pax3 and Myf5 are upstream factors of MyoD that are involved in muscle development, as evidenced by studies of mouse embryos^61^. Pax3 is initially expressed in the forming somite, but its expression is downregulated during specification in the sclerotome, whereas it remains expressed in the dermomyotome^62^.

Sonic hedgehog (Shh) is secreted from the notochord and floor plate of the neural tube^63^. Studies with mouse and bird embryos demonstrate that Shh functions as a crucial molecule in sclerotome formation^62,63^. *Shh* mutant mice lacked vertebral columns, and only a few rudimentary rib cartilages were formed^64^. Mouse embryos with deletion of both *Gli2* and *Gli3*, the downstream factors of *Shh*, exhibited severely reduced expression of *Pax1* and *Pax9*; further, *Sox9* expression was undetectable in somites^65^. However, *Shh*-null mice still showed transient Pax1 expression. These results imply that Shh is a crucial inducer for Pax1, Pax9, and Sox9 through Gli2 and Gli3^65^, although other signals may also be involved in the induction^64^. Studies in mice and birds showed that Noggin was expressed in the node, notochord, and dorsal somite and that it inhibited BMP4 activity during sclerotome specification^63,66^. Shh also competed with Wnt signaling from the roof plate and surface ectoderm, and Wnt functioned in these locations to maintain the somite epithelial state and induce the dermomyotome in chick embryos^62^. Collectively, these results show that high levels of Shh activation and low levels of Wnt and BMP signaling are required to determine sclerotomal fate.

Pax1 and Pax9, transcription factors of the Pax family, are specifically expressed in the large part of sclerotomes. Homozygous *Pax1*-null newborn mice showed severe abnormalities in the axial skeleton^67^. On the other hand, homozygous *Pax9* mutant mice showed skeletal defects in the limbs and the skull, but exhibited no obvious defects in the axial skeleton^68^. In addition, *Pax1/Pax9* double mutant mice showed much more severe phenotypes than *Pax1* single homozygous mutants; *Pax1/Pax9* double mutants completely lack tissues derived from the medial part of the sclerotome, such as the vertebral bodies, the intervertebral discs, and the proximal parts of the ribs^69^. The condensation of the ventromedial sclerotome around the notochord was also prevented in the double mutants, resulting in an impairment of chondrogenesis and vertebral formation^69^. Moreover, a rescue experiment in mice showed that Pax1 compensated for Pax9 function, whereas Pax9 did not compensate for Pax1 function^69^.

Nkx3.2 (Bapx1) is a homeobox-containing transcription factor expressed in the sclerotome during early embryonic mouse development^70^. A targeted disruption of the *Nkx3.2* gene in mice resulted in lethal skeletal dysplasia with abnormal development of the vertebral column and craniofacial bones^70^, and a failure of cartilage development with decreased expression of Sox9 and type II collagen^69^. In addition, condensation of sclerotomal cells in the vertebral anlagen around the notochord was completely lost during early embryogenesis in *Nkx3.2* mutant mice^71^. Analyses of mouse embryos with a double mutation of *Pax1/Pax9* revealed that Nkx3.2 expression in the sclerotome required the presence of both Pax1 and Pax9 in a dose-dependent manner^72^. In the same study, Nkx3.2 was found to be induced by the overexpression of Pax1 even without Shh. Furthermore, Pax1 and Pax9 transactivated the *Nkx3.2* promotor through direct interaction with DNA, indicating that *Nkx3.2* is a direct target of Pax1 and Pax9^72^. These results suggest that Nkx3.2 functions downstream of Pax1 and Pax9, and plays crucial roles in chondrogenesis and vertebral development^71^.

Meox1 and Meox2 are essential for somite formation, as described above, and they contribute to somite development. *Meox1/2* double mutant mice lacked Pax1 expression in the paraxial mesoderm, and they exhibited an attenuation of Pax3 and Pax7 expression, resulting in a failure of dermomyotome differentiation^34^. These deficiencies led to abnormalities in the axial skeleton, such as a lack of normal vertebral columns^34^, as well as major defects in the development of the somite-derived skeletal musculature^34^. In addition, the expression of Nkx3.2 in mice with mutations in both *Meox1* and *Meox2* was much more severe than it was in mice with a single *Meox1* mutation^34^. These results suggest that Meox1 and Meox2 coordinate with and compensate for each other during sclerotome and dermomyotome development.

### Subsets of the sclerotome

Because of its location, the sclerotome is in contact with various cell populations that produce different signaling molecules, resulting in the establishment of various subpopulations along the ventral–dorsal, medial–lateral, and anterior–posterior axes^12^. These conclusions were drawn mainly from studies on birds.

The center part of the sclerotome forms the mesenchymal core of the epithelial somite, which mainly contributes to the formation of intervertebral discs and joints of the vertebral column^73^. Thus, Christ named this sclerotomal subdomain the “arthrotome”^12^. The dorsal part of the sclerotome is located close to the myotome. FGF ligands, such as Fgf8, are secreted from the myotome and induce *scleraxis* expression^74^. These signaling activities give rise to the syndetome population, which is a precursor of the vertebral tendons and ligaments^74^. In the dorsomedial and lateral parts of the sclerotome, *Pax1* expression is downregulated by BMP4 from the dorsal neural tube, intermediate mesoderm, and lateral plate mesoderm, leading to the development that is independent of notochordal signals^75,76^. The dorsomedial part of the sclerotome forms the spine and arch, whereas the lateral part of the sclerotome forms the distal ribs^75^. This depends on Bmp4 secreted from the dorsal neural tube and lateral plate mesoderm^75^. Moreover, these two parts are devoid of Pax1 expression^75^. The medial sclerotome located adjacent to the lateral surface of the neural tube was identified, as a population giving rise to the blood vessels and meninges of the spinal cord^77^. In response to signaling molecules secreted from the notochord, the ventromedial sclerotome strongly expresses *Pax1* and migrates medially toward the notochord. These cells form the perinotochordal tube, which gives rise to the vertebral bodies and intervertebral discs^78^. Last, the ventral–posterior sclerotome with endothelial precursor potential was recently named the endotome^13,79^. This part of the sclerotome migrates and differentiates into vascular cells of the dorsal aorta and intervertebral blood vessels, as shown in studies with chicks^79,80^.

## Induction of mesodermal populations in vitro (Figs. 2 and 3)

By utilizing knowledge of mesoderm development, researchers have attempted to recapitulate the in vivo development processes using in vitro settings. Next, we will summarize key reports about these in vitro models, and there will be a particular focus on studies, using PSCs from mice and humans.

**Fig. 2:**
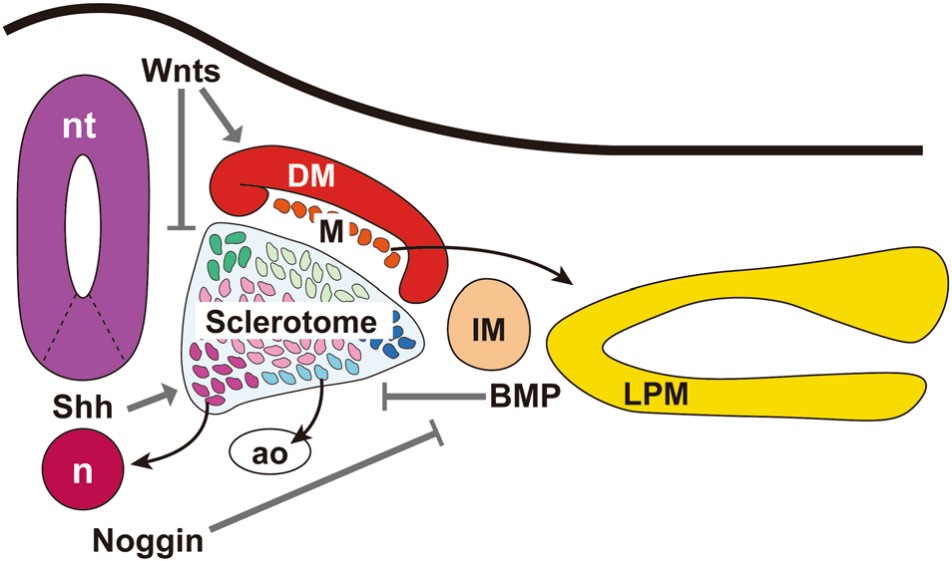
Schematic of the relationship between the sclerotome and other tissues. A large part of the sclerotome is induced depending on Shh signaling from the notochord and floor plate. Shh also competes with Wnt, which induces the dermomyotome and maintains the epithelial state in somites. The notochord also produces Noggin, which inhibits BMP signaling and supports sclerotome induction^63^. nt neural tube, n notochord, ao dorsal aorta, DM dermomyotome, M myotome, IM intermediate mesoderm, LPM lateral plate mesoderm.

### Presomitic mesoderm induction from PSCs via the primitive streak and neuromesodermal progenitor

Following the revelation of the pivotal roles of Wnt signaling in development, previous reports showed that Wnt activators, such as CHIR99021, a GSK3 inhibitor, induced cell types of the primitive streak and neuromesodermal progenitor from mouse and human PSCs in vitro^30,81–85^. In addition, several papers showed that combinatorial activation of Wnt and FGF signaling enhanced the induction efficiency of the primitive streak and neuromesodermal progenitors^81–83,85–87^. The requirements for TGFβ signaling differ among the differentiation stages. Nodal, a TGFβ activator, has been shown to enhance induction of the anterior primitive streak, which gives rise to neuromesodermal progenitors^27,82,85,88^. In contrast, the combination of a TGFβ inhibitor and a Wnt activator enhanced induction of the presomitic mesoderm, which possibly occurred through induction of the primitive streak^83,86^. Given that Wnt activation may be a trigger for endogenous FGF and Nodal activation in vitro^83,85,89,90^, Wnt activation is likely sufficient. FGF and Nodal may accelerate Wnt-mediated cell fate commitment or differentiation in the primitive streak and neuromesodermal progenitors.

**Fig. 3:**
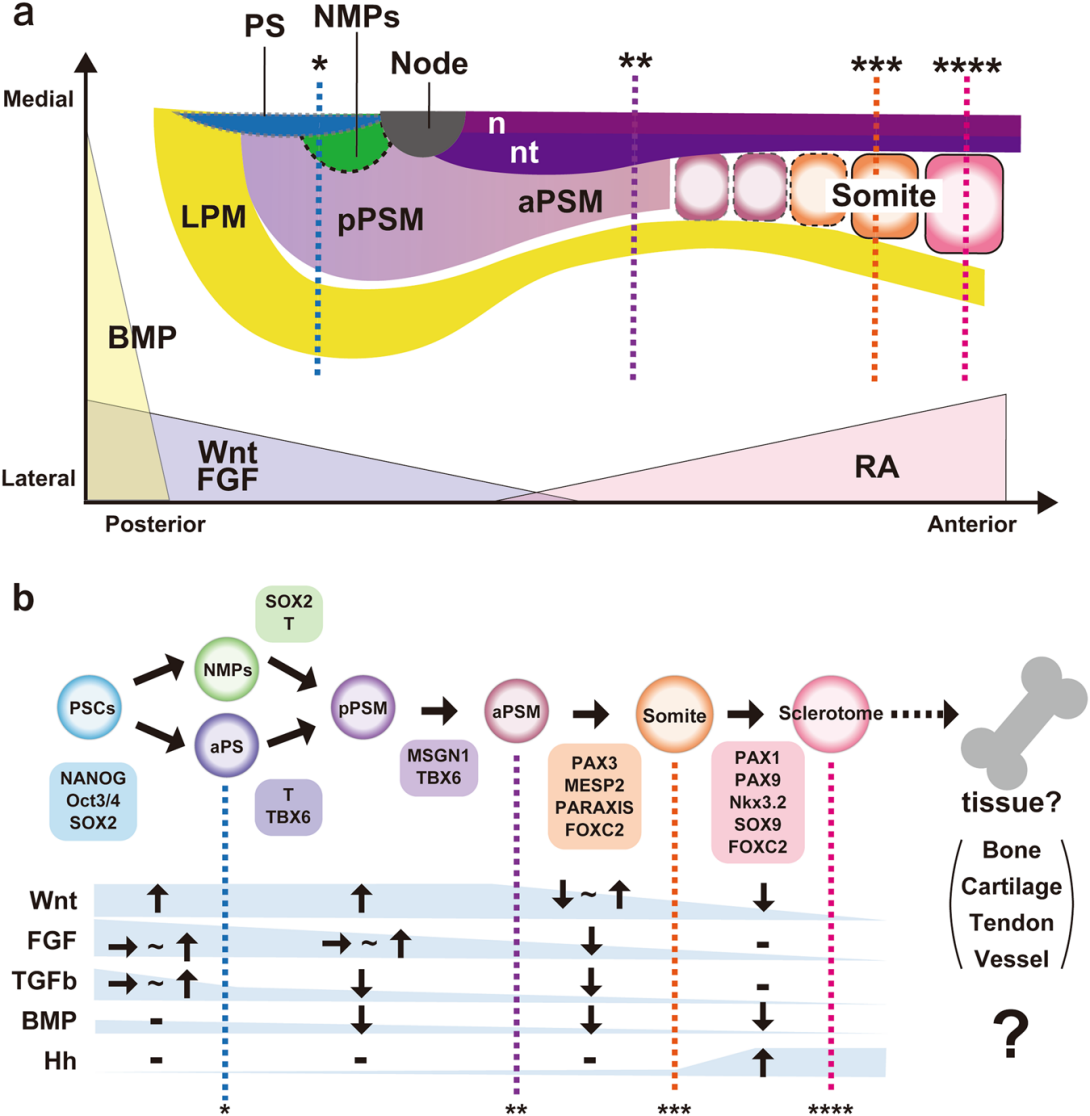
Flowchart of paraxial mesodermal development and sclerotome specification. **a** A dorsal view of the mesoderm fate in the posterior of an amniote embryo. The paraxial mesoderm forms in the primitive streak and from NMPs. Through the elongation of the embryo axis, the PSM is located from posterior to anterior according to the body axis produced by competing gradients of Wnt, FGF, and RA. The anterior PSM forms somite at the determination front depending on the FGF gradient and Notch signaling. **b** A flowchart of the in vitro differentiation process from PSCs to sclerotomes, with critical inducers and specific markers in each stage. In the future, a next step will be to differentiate the induced sclerotome into sclerotome derivatives, such as bone, tendon, and vessels. RA retinoic acid, NMP neuromesodermal progenitor, PS primitive streak, aPS anterior primitive streak, pPSM posterior presomitic mesoderm, aPSM anterior presomitic mesoderm, nt neural tube, n notochord, LPM lateral plate mesoderm. Asterisks in each of the differentiation stages in **a** correspond to the stages highlighted by asterisks in **b**.

In the differentiation from the primitive streak/neuromesodermal progenitor into the presomitic mesoderm, activation of Wnt signaling with CHIR was reported to promote paraxial mesoderm cell fate, and to upregulate Tbx6 and Msgn1 expression^82,83,86,91^. The combination of a BMP inhibitor, LDN-193189, and CHIR improved the induction efficiency of the paraxial mesoderm^82,83,85–87,89^, possibly because the fate of mesodermal progenitors can be changed from the paraxial mesoderm to the lateral plate mesoderm according to the gradient of BMP activity. Conversely, exogenous BMP activation promoted lateral plate mesoderm fate, but suppressed paraxial mesoderm fate^82^.

One difference has been observed between humans and other model organisms during paraxial mesoderm differentiation in vivo: TGFβ signaling was downregulated during presomitic mesoderm induction in humans, but not in animals^83^. Consistent with this finding, some reports showed that the combination of a TGFβ inhibitor with a BMP inhibitor increased the efficacy of the induction from human PSCs to the paraxial mesoderm or anterior presomitic mesoderm/somite^82,83,85–87^.

### Somite induction and sclerotome specification

As described earlier, the anterior presomitic mesoderm in the determination front forms somites according to the oscillation of Notch signaling and the antagonizing effects of Wnt/FGF. Some reports have tried to recapitulate this signaling transition in vitro by removing Wnt and FGF/ERK signaling effects^82,85,89^. However, activation of Wnt signaling, regardless of the presence or absence of FGF, was sufficient to recapitulate this transition in both mouse and human PSCs^86,91^. Molecular patterns similar to the in vivo segmentation program were observed in hPSCs^82,86,89,91^: downregulation of *Msgn1*, transient upregulation of *Mesp2*, and induction of *Pax3*^82,85–87,89,91^. These findings suggest that once the cells respond to those key signaling factors, they are set to undergo the sequences of differentiation in a cell autonomous manner.

The BMP gradient is a critical factor in the determination of the mediolateral axis during mesoderm development and in somite specification^10,63^. In line with this, some reports showed that a BMP inhibitor improved somite–sclerotome induction, possibly by both promoting medial fate and protecting the mesodermal population from lateralization^10,82–86^.

For sclerotome induction, the activation of Hh signaling seems the most important^62,64,65^. Although various protocols were established for sclerotome induction, all of them used Hh agonists^82–84,86^. This is due to the indispensable roles of Shh in the developmental stage, which were demonstrated by several studies in mice and birds: Shh is produced by the notochord and floor plate; it is essential for somite specification into the sclerotome in vivo^62,63^. In addition, the combination of an Hh agonist with a Wnt inhibitor recapitulated the competing relationship of Shh and Wnt^62,82^; the combination of an Hh agonist with a BMP inhibitor recapitulated Noggin activity to inhibit BMP signals from the intermediate mesoderm or lateral plate mesoderm, and to promote sclerotome differentiation^83–86^. In any case, the cell fate commitment to the sclerotome was achieved by the expression of specific transcription factors, such as PAX1, PAX9, SOX9, and NKX3.2^82–86^. Based on what has been learned about mouse and bird embryo development^63,66^, the combination of Hh signaling and BMP inhibition may be sufficient for sclerotome induction.

Two concerns that remain to be resolved with respect to in vitro differentiation are (1) heterogeneity of the induced cells and (2) differences in differentiation protocols among species. First, as described above, although several protocols were established to induce paraxial mesoderm derivatives from PSCs, there is no protocol that can exclusively induce generation of the desired population and exclude unwanted populations. The remaining undifferentiated cells or wrongly differentiated cell populations can cause oncogenicity, which leads to considerable risk in a clinical context. Heterogeneous populations may also cause less efficiency of the induction of wanted cell populations. Thus, manipulating the activities of combinatorial signaling pathways in a stepwise manner will be required not only for proper modeling of development, but also for clinical usage. Second, recent studies have shown divergence between the developmental process of human and mouse embryos^83,92^. This is consistent with in vitro studies that show that an optimized differentiation protocol to induce a cell type in mice is not always optimal for induction of the same cell type in humans; different combinations of signaling activities are required for different species to some extent^83,93^. Thus, further comparative analysis among different species will provide clues for establishing an optimized protocol for the differentiation of human PSCs into specific cell types.

## Future perspectives

As we described in this review, skeletal elements are derived from the neural crest, lateral plate mesoderm, and paraxial mesoderm. How does the difference in origin affect the properties of skeletal cell types in terms of their differentiation efficacy and regenerative capacity? Because protocols for the differentiation of skeletal cells through the neural crest and lateral plate mesoderm were recently proposed^94,95^, it may be possible to perform such comparative analyses in the future. This will provide insights into the origin-distinct process of skeletal development and will generate a strategy of skeletal regeneration.

As we have shown in the above review, the current stepwise protocols enable the generation of sclerotome cell types. Recent studies have further established protocols that recapitulate the segmentation clock in mouse gastruloids^96^, and in the somite induced from human and mouse PSCs^85,87^. The next step will be further optimization of these protocols to induce sclerotome derivatives, including bone, cartilage, and tendon. Some groups have proposed protocols to induce osteoblasts, chondrocytes, and tenocytes from mouse or human PSCs via the sclerotome^82,83,85,86^.

Previous reports have established protocols for directing the differentiation of PSCs toward three-dimensional organoids of various tissues, such as retina^97^ and limb bud^95^. In addition, some of these protocols have already been applied in clinical trials^5^. In contrast, it remains challenging to generate three-dimensional skeletal tissues even though we can induce every cell component of the tissue, including osteoblasts and chondrocytes. To do this, we may need not only a deeper understanding of skeletal development, but also tissue engineering approaches that take advantage of biomaterials. We recently reported that differentiation of osteoblasts from mouse ES cells with a three-dimensional atelocollagen scaffold enhanced osteoblast maturation^98^. Several biomaterials and three-dimensional culture systems have been developed^95,99^, although further optimization is needed. In addition to enabling the modeling of skeletal development, the three-dimensional system will provide a model that recapitulates bone metabolism under physiological conditions: remodeling by coupling of bone-forming osteoblasts and bone-resorbing osteoclasts, and responses of the cells upon mechanical stresses. The three-dimensional system will thus be useful for drug screening and disease modeling.

For clinical settings, the transplantation of human PSC-derived tissues is one of the goals in translational studies, but several issues remain to be solved—namely, the quality, quantity, safety, and availability of the resulting tissues. Thus, when we implant tissues derived from PSCs into patients, the tissues that are produced must meet several criteria: they must have sufficient function, sufficient size or volume, little or no immune response, and no oncogenicity. Researchers have tried to satisfy these criteria by using iPS cells derived from the patients themselves or from donors with the same haplotype of HLA^100^, reducing or removing xenogeneic components, such as fetal bovine serum and Matrigel^101^, and removing undifferentiated iPS cells by a specific reagent^102^. Further clinical studies, especially those focusing on safety, will be needed in the future.

In addition, several attempts have been made to utilize human PSCs in clinical settings. Disease modeling and drug discovery are attractive applications for the treatment of various diseases. Recent reports have presented various models of skeletal diseases using patient-derived iPS cells, such as models of fibrodysplasia ossificans progressiva, FGFR3-related skeletal dysplasia, and osteogenesis imperfecta. The pathological features of these diseases were recapitulated well by chondrocytes and osteoblasts differentiated from patient-derived iPS cells; accordingly, these differentiation platforms have been utilized for drug screening^4,86,103,104^. In addition, some reports have demonstrated human PSC-derived skeletal stem/progenitor cells and mesenchymal stem/stromal cells that have multipotent capacity to differentiate into several lineages of skeletal components^105,106^. These cells may be valuable cell sources for cell therapies for skeletal diseases. Taken together, these findings show that the potential applications for human PSCs have been expanded in both basic science and clinical medicine. An improved understanding of the development of paraxial mesoderm derivatives and further optimization of the protocols for the differentiation of human PSCs into skeletal tissues will provide both insights into human skeletal development and valuable tools for clinical settings.
